# Mask-Point: Automatic 3D Surface Defects Detection Network for Fiber-Reinforced Resin Matrix Composites

**DOI:** 10.3390/polym14163390

**Published:** 2022-08-19

**Authors:** Helin Li, Bin Lin, Chen Zhang, Liang Xu, Tianyi Sui, Yang Wang, Xinquan Hao, Deyu Lou, Hongyu Li

**Affiliations:** 1 School of Mechanical Engineering, Tianjin University, Tianjin 300072, China; 2 Science and Technology of Advanced Functional Composite Laboratory, Aerospace Research Institute of Materials and Processing Technology, Beijing 100076, China; 3 International Institute for Innovative Design and Intelligent Manufacturing, Tianjin University, Shaoxing 312000, China

**Keywords:** fiber-reinforced resin matrix composites, composite materials, surface defect, defect detection, semantic segmentation, automated optical inspection

## Abstract

Surface defects of fiber-reinforced resin matrix composites (FRRMCs) adversely affect their appearance and performance. To accurately and efficiently detect the three-dimensional (3D) surface defects of FRRMCs, a novel lightweight and two-stage semantic segmentation network, i.e., Mask-Point, is proposed. Stage 1 of Mask-Point is the multi-head 3D region proposal extractors (RPEs), generating several 3D regions of interest (ROIs). Stage 2 is the 3D aggregation stage composed of the shared classifier, shared filter, and non-maximum suppression (NMS). The two stages work together to detect the surface defects. To evaluate the performance of Mask-Point, a new 3D surface defects dataset of FRRMCs containing about 120 million points is produced. Training and test experiments show that the accuracy and the mean intersection of union (mIoU) increase as the number of different 3D RPEs increases in Stage 1, but the inference speed becomes slower when the number of different 3D RPEs increases. The best accuracy, mIoU, and inference speed of the Mask-Point model could reach 0.9997, 0.9402, and 320,000 points/s, respectively. Moreover, comparison experiments also show that Mask-Point offers relatively the best segmentation performance compared with several other typical 3D semantic segmentation networks. The mIoU of Mask-Point is about 30% ahead of the sub-optimal 3D semantic segmentation network PointNet. In addition, a distributed surface defects detection system based on Mask-Point is developed. The system is applied to scan real FRRMC products and detect their surface defects, and it achieves the relatively best detection performance in competition with skilled human workers. The above experiments demonstrate that the proposed Mask-Point could accurately and efficiently detect 3D surface defects of FRRMCs, and the Mask-Point also provides a new potential solution for the 3D surface defects detection of other similar materials

## 1. Introduction

Fiber-reinforced resin matrix composites (FRRMCs) have a series of advantages, such as high specific strength and modulus, so FRRMCs are widely used in aerospace, shipbuilding, and other industrial fields [1,2]. In the manufacturing process of FRRMCs, there are always tiny concave-shaped defects on their surface. The defects adversely influence the material’s appearance and performance. Recently, product quality problems and safety accidents caused by composite materials’ defects have been reported [3,4]. A batch of Boeing 787 airliners was recalled due to minor defects in the FRRMCs on the surface of the fuselage in 2020, which caused significant economic losses [5]. Therefore, it is significant to study the surface defects of the FRRMCs in depth. Many researchers have tried to reveal the generation mechanism of defects and reduce defects [6]. The formation of defects can be partly controlled by process parameters such as consolidation pressure, resin velocity, vacuum pressure, and cure temperature [7]. However, there are still quite a few surface defects that are difficult to eliminate. An effective way to achieve defect-free manufacturing is to detect surface defects during the manufacturing process and then fill the defects in the subsequent repairing process. The surface defects are mainly recognized by human workers through their visual detections using naked eyes or magnifiers nowadays. However, surface defects of FRRMCs are generally quite tiny, with feature sizes of about 0.2 to 3 mm, and the texture of the fibers under the surface may also lead to disturbances; thus, visual detections are actually challenging tasks even for skilled human workers. So, automated methods for detecting surface defects of FRRMCs are needed.

In the field of defects detection, major efforts have been devoted to 2D image-based object detection or segmentation methods [8,9,10]. However, 2D image-based results cannot directly provide the depth information of defects, so subsequent quantitative filling processes cannot be carried out effectively. As a result, many researchers have focused on 3D point cloud-based methods, and those methods can be divided into two main groups: (1) traditional methods and (2) deep learning-based methods. Traditional methods mainly take advantage of local geometric features such as the normals or curvatures, and thresholding methods are used to remove points of backgrounds and obtain points of defects [11,12,13]. Traditional methods are relatively intuitive and effective; however, the defection performance may be relatively poor if the segmentation threshold is chosen improperly, so their robustness and applicability deserve further improvement. As for the deep learning-based methods, the defects detection tasks can be treated as 3D semantic segmentation problems in deep learning. PointNet [14], PointNet++ [15], and Point transformer [16] are typical 3D semantic segmentation networks. However, the segmentation performance (mIoU) is still less than 0.75 on public datasets [17], even with the recent state-of-the-art semantic segmentation network. Their actual performance on defects detection also needs to be improved. As a result, more accurate 3D point cloud-based surface defects detection methods are urgently required, which are of practical significance for the FRRMC manufacturing process.

In this paper, defects detection is also treated as a special 3D semantic segmentation task. To accurately detect FRRMCs’ surface defects, a novel lightweight and two-stage 3D semantic segmentation network, i.e., Mask-Point, is proposed. The network mainly exploits the geometric features of defects and combines the advantages of traditional and deep learning methods. The main novelties and contributions of this research are (1) new multi-head 3D RPEs in Stage 1 of Mask-Point are designed for generating several 3D ROIs, allowing subsequent networks to focus on potential regions of defects, and (2) the shared classifier and NMS in Stage 2 of Mask-Point are designed for classifying and aggregating 3D ROIs to improve the overall segmentation performance.

## 2. Materials

### 2.1. The Manufacturing Process of FRRMC Products

**Figure 1:**
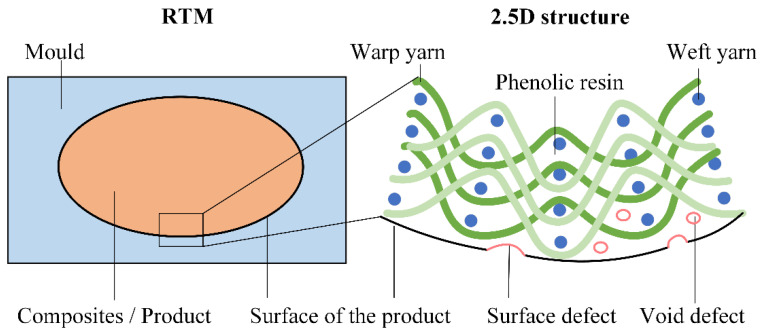
RTM process and the structure of 2.5 D quartz fiber-reinforced phenolic resin composites.

To illustrate the methodology of this paper, a representative FRRMC material, the 2.5D Quartz fiber-reinforced phenolic resin composite, was selected. It is a new composite material with superior overall performance [18]. As shown in Figure 1, the material is made of a 15 mm thick 2.5D quartz fiber fabric (provided by Nanjing Glass Fiber Research and Design Institute, Nanjing, China) and phenolic resin (provided by Beijing Glass Research and Design Institute, Beijing, China) through the resin transfer molding process (RTM) under atmospheric pressure. In the RTM process, the condensation reaction of phenolic resin curing will produce water. The water will be discharged as vapor because the curing temperature is controlled at 140 °C. In addition, alcoholic solvents in the phenolic resin will also escape from the matrix as gas due to the curing temperature. The above reasons lead to inner void defects and surface defects in the RTM process [19].

**Figure 2:**
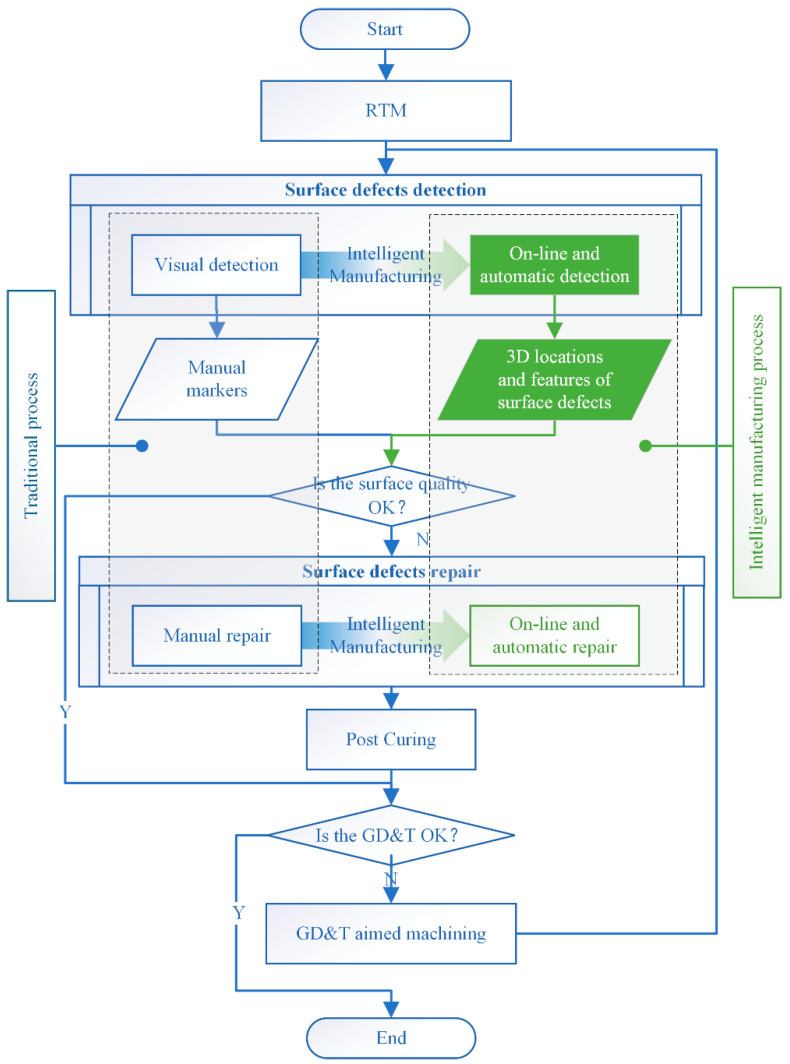
The traditional manufacturing process of FRRMC products and novel intelligent manufacturing solutions with automatic surface defects detection and repair process.

Since defects on the product’s surface will affect its appearance and performance, the surface quality standard for the product requires that there should be no surface defects with feature sizes of more than 0.2 mm. So, there are subsequent processes after the RTM. In the traditional manufacturing process, as shown in Figure 2, skilled workers detect surface defects using their naked eyes and magnifiers, then manually fill the surface defects with epoxy resin, and then the repaired product will be cured again. If the geometric dimensions and tolerances (GD&T) do not meet technical requirements, GD&T-aimed machining is also required subsequently. The above processes are looped until the surface quality meets factory standards.

However, it is not easy to significantly improve the efficiency and accuracy of human-dependent detection and repair for mass production. The integration of automatic inspection and analysis processes into the manufacturing process has been promoted by Industry 4.0 [20] and Intelligent Manufacturing [21,22,23] in recent years. Therefore, automatic inspection and surface defect detection are the main objectives of this study. The automatic detections system also outputs quantitative information about the surface defects and provides technical support for the subsequent automatic repair process in the future.

### 2.2. Defects of FRRMCs

**Figure 3:**
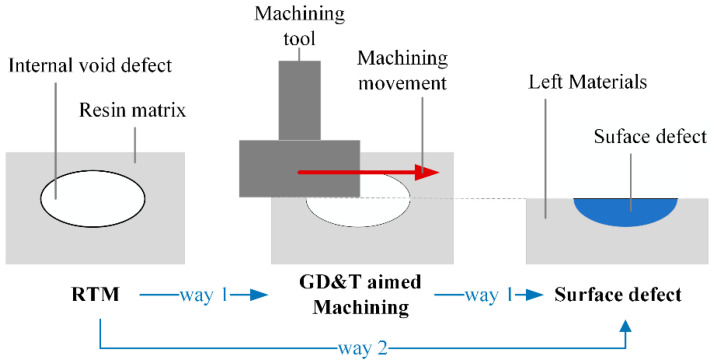
Formation of surface defects: way 1 is the machining revealed defects, and way 2 is directly formed defects in RTM.

The surface defects of the FRRMCs in this study do not only come directly from the RTM (see way 1 in Figure 3). If the GD&T of a product does not meet technical requirements, machining will be performed. Thus, portions of internal void defects will be removed, resulting in machining revealing defects.

Surface defects are classified into four categories in ISO 8785:1998 [24]: (a) recession; (b) raising; (c) combined surface imperfections; (d) area imperfections, appearance imperfections. Most void defects are ellipsoidal in shape due to air pressures, so most machining-revealed defects are localized in the ellipsoid. Most directly formed defects in RTM have irregular shapes. However, all defects are recessed concerning their surrounding surface. Therefore, the surface defects of FRRMCs belong to the recession category. The location and number of surface defects are highly random, which brings challenges to the goal of automatic detection.

**Figure 4:**
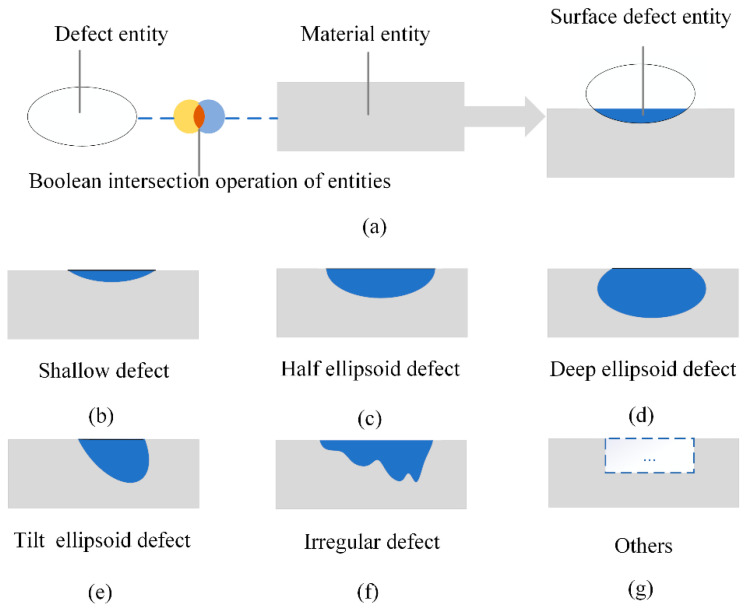
Three-dimensional computer graphics analysis of surface defects. (**a**) The surface defect entity is a Boolean intersection of the defect entity and material entity; (**b**) shallow defect; (**c**) half ellipsoid defect; (**d**) deep ellipsoid defect; (**e**) tilt ellipsoid defect; (**f**) irregular defect; (**g**) other shapes of defects.

Treating both the defect and the material as solid entity models helps to quantitatively evaluate surface defects in 3D computer graphics effectively. A defect can be considered as a Boolean intersection of the defect entity and material entity, as shown in Figure 4. Thus, the combination of different values of the following factors can be applied to represent numerous defects in the real physical world: the 3D shape and size of the defective entity, the 3D shape and size of different material entities, the relative position and relative attitude of the defective entity and the material entity in space, etc. Several kinds of defects in engineering can be derived according to the above rules quite easily, as shown in Figure 4b–g; such a classification is intuitive, but it is still highly subjective. ISO 8785:1998 provides several features of surface defects to characterize surface defects, such as SIM*_l_*, SIM*_w_*, SIM*_a_*, etc. [24]; therefore, surface defects can be evaluated with the same standard metrics rather than be classified subjectively. So, the following features are designed to characterize surface defects of FRRMCs according to ISO 8785:1998 [24]:

(a) SDL, surface defect length. The maximum size of the surface defect measured parallel to the reference plane.
(b) SDW, surface defect width. The maximum size of the surface defect measured parallel to the reference plane and perpendicular to the length of the surface defect.
(c) SDD, surface defect depth. The distance between the reference surface and the lowest point in the surface defect measured vertically from the reference surface.
(d) CSDA, surface defect area. Area of the surface defect.
(e) SDV, surface defect volume. The volume of the surface defect envelope.

## 3. Methods

### 3.1. Data Acquisition

**Figure 5:**
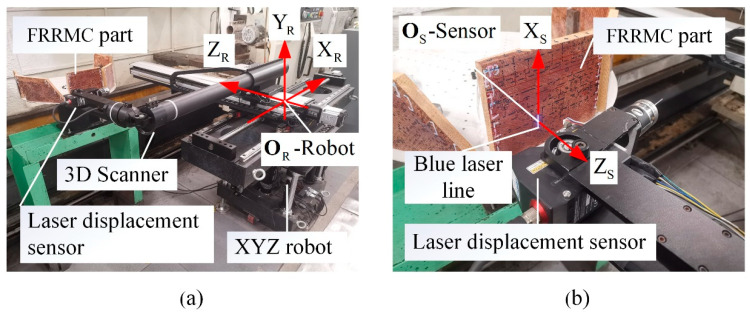
Data acquisition of FRRMCs’ surface defects with a self-developed 3D laser scanner. (**a**) A 3D laser scanner that is composed of an XYZ robot and a 2D laser displacement sensor; (**b**) the laser displacement sensor (KEYENCE LJ-V7060) and an FRRMC part.

In order to meet the requirements of online FRRMCs’ surface defects detection and obtain high-quality input data for the methods in this paper, a multifunctional 3D laser scanner is developed, as shown in Figure 5a; the scanner consists of an XYZ robot and a 2D laser displacement sensor mounted on the end flange of the robot. The main technical specification of the robot is shown in Table 1. The laser displacement sensor is a KEYENCE LJ-V7060 with relatively high accuracy and repeatability, and its technical specification is shown in Table 2 [25]. The interval between points on a measured profile from the sensor is 20 μm, which is one-tenth of the defect’s smallest feature size, and the trigger interval is as fast as 16 μs, so the 3D scanner can capture tiny details of the measured surface even with a relatively high scanning speed. In addition, the laser displacement sensor uses the blue laser with a wavelength of 405 nm. The blue laser has better focusing characteristics and anti-interference properties [25], making the measured results almost unaffected by the internal texture below the surface, allowing the scanner to obtain more accurate results than 2D image-based instruments. As a result, the 3D laser scanner provides powerful technical support for surface defects detection in this paper.

**Figure 6:**
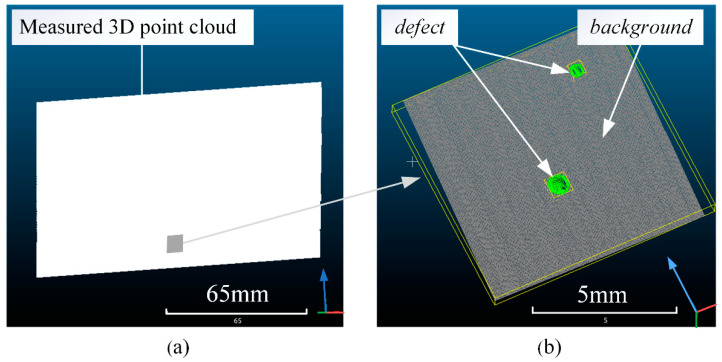
Dataset of FRRMCs’ surface defects. (**a**) A measured 3D point cloud; (**b**) a point cloud sample and its labels.

The FRRMC test parts are fixed on the fixture; then, the 3D scanner scans them one by one. Thus, the original 3D point clouds are obtained; one of the originally measured 3D point clouds is shown in Figure 6a. Next, every original 3D point cloud was sub-sampled to keep the distance of 0.02 mm between points. In order to improve the inference speed and facilitate parallel computing during training and test, the original point clouds are divided into smaller sub-point clouds into cubic spaces with an edge length of 8 mm. Each sub-point cloud contains about 160,000 points. Then, with the help of manual geometric analysis and label software, such as CloudCompare [26], each point was labeled as either “background” or “defect” category, as shown in Figure 6b. Finally, a dataset with 120 million points and a total of 749 samples was generated. These samples actually include surface defects from different RTM parameters and are therefore representative. Among the 749 samples, 249 samples formed the test set, and the remaining 500 samples formed the training set. The data augmentation techniques, such as random space clipping, rotation, and shift noise [15], are applied to the training set, so the training set is expanded by six times, i.e., 3000 copies.

### 3.2. Mask-Point Based Defects Detection

**Figure 7:**
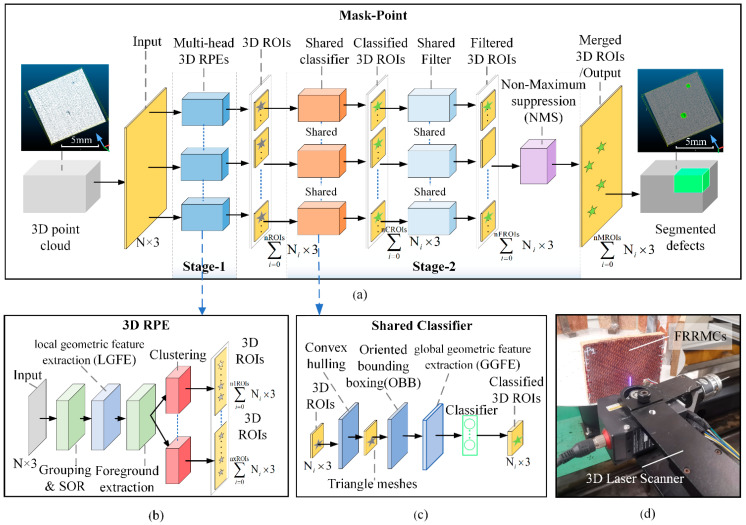
The architecture of Mask-Point. (**a**) The architecture of Mask-Point; (**b**) the structure of a 3D RPE; (**c**) the structure of the shared classifier; (**d**) scanning of FRRMCs.

We propose a novel Mask-Point to detect FRRMCs’ surface defects. The Mask-Point is a lightweight, two-stage semantic segmentation network especially designed for the 3D surface defects detection task. As shown in Figure 7, Stage 1 of Mask-Point is the multi-head 3D RPEs, generating several 3D ROIs. Stage 2 of Mask-Point is an aggregation stage composed of the shared classifier, shared filter, and non-maximum suppression (NMS). Finally, the segmented results can be produced at the end of Stage 2.

#### 3.2.1. Stage 1 of Mask-Point: Multi-Head 3D RPEs

The surface defects of FRRMCs are recessed relative to the surrounding regions, which could be characterized by the difference in local geometric features between defect points and their neighboring points. Therefore, Stage 1 of Mask-Point is designed, as shown in Figure 7a,b. The 3D point cloud data were firstly input into the grouping and SOR layer. The grouping operator gathers the nearest neighboring points of every single point, and the SOR operator is a statistical outlier removal filter to reduce noises. Then, the local geometric feature extraction (LFGE) layer extracts the local geometric features, such as Gaussian curvature or curvature change rate (NCR). In the case of NCR, if there is a neighborhood point set ($S_{i}$) with three principal components of the covariance matrix ($\lambda_{i1}$, $\lambda_{i2}$, $\lambda_{i3}$, and $\lambda_{i1}>\lambda_{i2}>\lambda_{i3}$). ${\mathrm{NCR}}_{i}$ can be calculated with Equation (1).

(1) $${\mathrm{NCR}}_{i}=\frac{\lambda_{i1}}{\lambda_{i1}+\lambda_{i2}+\lambda_{i3}}$$

**Figure 8:**
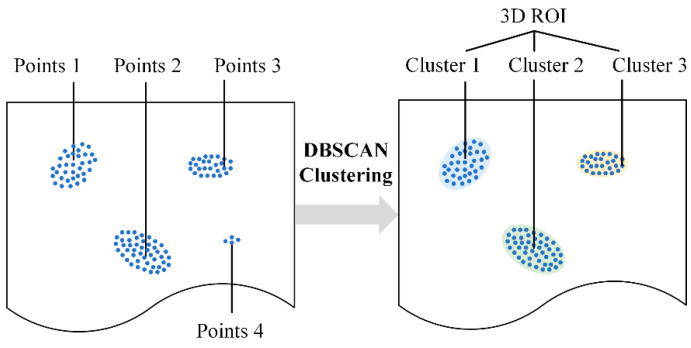
Effects of the DBSCAN clustering algorithm and the formation of 3D ROIs.

Then, the foreground extraction (FE) layer extracts the foreground points by a quantile filter, and the clustering layer is used to generate several 3D ROIs. The density-based spatial clustering of application with noise (DBSCAN) algorithm is introduced in the clustering layer. DBSCAN can efficiently discover similarities and differences in data and determine any shape of clusters that may exist in a given dataset without any a priori knowledge and human intervention. These capabilities make it an ideal solution for aggregating defects. Most points with insignificant local geometric features will be filtered out, leaving only the point clouds of potential defect regions through LGFE and FE layers. We assume that the left points are distributed like Points 1–4 in Figure 8. Some points (Points 1–3) will be clustered in three clusters by DBSCAN and become 3D ROIs (Clusters 1–3) in Figure 8 because the points meet the requirements of DBSCAN to form clusters. Points 4 cannot form a cluster because they do not satisfy the conditions of DBSACN to form a cluster.

The pseudo-code of DBSCAN is presented in Algorithm 1 [27]. DBSCAN has only two hyperparameters: the cluster’s radius (eps) and the minimum number of points within a sphere of radius eps (minpts). When given a set of points in space, DBSCAN starts with a core coordinate and continuously expands it to a region where the density is reachable, resulting in a maximized region (called a cluster) containing core and boundary coordinates. DBSAN has high computational efficiency, but it may be sensitive to its hyperparameters [28] and may perform poorly on multi-density data [29,30].
**Algorithm 1**: DBSCAN clustering**Input**: eps, minpts, **X** **Output**: the set of clusters**1.** **2.** **3.** **4.** **5.** **6.** **7.** **8.** **9.** **10.** **11.** **12.** **13.** **14.** **15.** **16.** **17.** **18.** **19.** **20.** **21.** **22.** **23.** **24.** **25.** **26.****procedure** DBSCAN(**X**, eps, minpts) **for each** point P ∈ **X do**  **if** label(P) ≠ visited **then**   label (P) ← visited  N ← Neighbors (**P**, eps)  **if** N < minpts **then** mark P as Noise`  **else**   C ← P   **for each** point Pn∈N **do**    N ← N\Pn    **if** label (Pn) ≠ visited **then**     label (Pn) ← visited     Nn ← Neighbors(Pn, eps)     **if** Nn ≥ minpts **then**     N ← N ∪ Nn    **if** Pn not a member of any cluster **then**    C ← C ∪ Pn    **end if**     **end if**    **end if**   **end for**   **end if**  **end if** **end for** **end procedure**

The scanned data may be multi-density in actual scanning because the surface of the products may be curved even scanned uniformly. Thus, the scanned point clouds may be non-uniform. A single DBSCAN does not work for multi-density data, so multi-head 3D RPEs are proposed. Each 3D RPE can be equipped with a DBSCAN with specified parameters to produce one kind of 3D ROI. Multiple 3D RPEs with different DBSCAN and hyperparameters can thus handle multi-density data. In addition, the 3D RPE can accommodate different LGFE and FE layers to construct as many 3D ROIs as possible, thus increasing the probability of successfully detecting surface defects. Multi-head 3D RPEs can overcome the sensitivity to hyperparameters that occurred in traditional methods, improve the network’s robustness, and facilitate parallel computing.

#### 3.2.2. Stage 2 of Mask-Point: Aggregation Stage

Stage 1 of Mask-Point outputs several 3D ROIs, but not all 3D ROIs belong to the target surface defects. Some 3D ROIs may also overlap with each other, which is similar to the Mask-RCNN in 2D image segmentation [29,30]. Mask-RCNN firstly calculates the probability of ROIs through the classifier. Then, the NMS is applied to remove ROIs with lower probabilities to deal with the overlap problem and obtain the final detection results. A new 3D shared classifier and 3D NMS are proposed in this paper to deal with 3D ROIs in Stage 2, referring to the principles of Mask-RCNN.

**Figure 9:**
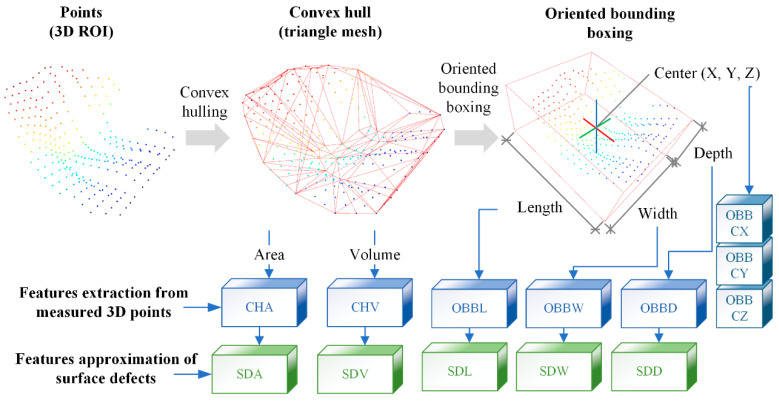
Feature extraction from 3D ROIs; the extracted CHA, CHV, OBBL, OBBW, and OBBD are applied to approximate surface defects’ features of SDA, SDV, SDL, SDW, and SDD.

The global geometric features SDL, SDW, SDD, SDA, and SDV mentioned in Section 2 are adopted to construct the shared classifier. Because the geometrical features of surface defects are distinguishable from non-defective ones, the geometric features are also required by the subsequent repair process in the manufacturing process of the FRRMC products. However, the outputs of Stage 1 are 3D ROIs with scattered points rather than features, so the pre-processing and feature extraction layers are required to extract geometric features. As shown in Figure 7c, a series of layers, including the convex hulling layer, triangle meshing layer, oriented bounding boxing layer, and the global geometric feature extraction (GGFE) layer, is designed in Stage 2 to extract the global geometric features. A detailed procedure is shown in Figure 9. The convex hulling layer computes the convex hull [31] of a 3D ROI and forms a triangle mesh, and then the SDA and SDV features can be approximated by the surface area (CHA) and volume (CHV) of the convex hull. The minimum bounding box (OBB) can be calculated in the oriented bounding boxing layer, and the length (OBBL), width (OBBW), and depth (OBBD) of OBB can be applied to approximate the SDL, SDW, and SDD features of a defect. Thus, all features of a surface defect can be approximated through computer graphics analyses.

The convex hull of a shape is defined as the intersection of all convex sets containing a given subset of Euclidean space. Quickhull is an efficient algorithm for the automatic computation of convex hulls for high-dimensional data [31]. It has the expected time complexity of *O*(*n*log*n*), but it could degenerate to *O*(*n*^2^) in the worst scenario, where *n* is the number of input points. The pseudo-code for Quickhull is shown in Algorithm 2 [31]. Each unprocessed point is first given a place in an outer set. Then, Quickhull constructs new outside sets from the outside sets of the visible facets to produce cones of new facets. One of the new facets is chosen when a point is above many new facets. The point is inside the convex hull and can be ignored if it is below all new facets. The procedure above additionally logs each outside set’s furthest point. For the initial simplex, Quickhull chooses a nondegenerate collection of points and chooses points with a minimum coordinate if possible. The convex hull can be represented as a triangular mesh, and its CHA and CHV can be extracted in the GGFE layer based on the triangular mesh, which is then used to characterize the SDA and SDV features of the surface defects. With the convex hull, the OBB of the convex hull can be computed by the PCA of the convex hull’s vertexes [32]. The OBBL, OBBW, and OBBD of OBB can be extracted in the GGFE layer, then applied to represent the surface defects’ SDL, SDW, and SDD features.
**Algorithm 2**: Quickhull algorithm for the convex hull**Input**: points **Output**: processed outside set (convex hull)**1.** **2.** **3.** **4.** **5.** **6.** **7.** **8.** **9.** **10.** **11.** **12.** **13.** **14.** **15.** **16.** **17.** **18.** **19.** **20.** **21.** **22.** **23.** **24.** **25.** **26.** **27.** **28.** **29.** **30.** **31.** **32.****procedure** Quickhull(points) create a simplex of *d* + 1 points **for each** facet F **do**  **for each** unassigned point p **do**   **if** p is above F **then**    assign p to outside set of F   **end if**  **end for** **end for** **for each** facet F with a non-empty outside set **do**  select the furthest point p of F’s outside set  initialize the visible set V to F  **for** all unvisited neighbors N of facets in V   **if** p is above N **then**    add N to V   **end if** **end for**  the boundary of V is the set of horizon ridges H  **for each** ridge R in H **do**   create a new facet from R and p   link the new facet to its neighbors  **end for**  **for each** new facet F’ **do**   **for each** unassigned point q in an outside set of a facet in V **do**    **if** g is above F’ **then**     assign q to outside set of F’    **end if**  **end for** **end for**  delete the facets in V **end for** **end procedure**

The geometric features, including CHA, CHV, OBBL, OBBW, and OBBD, are then input to the shared classifier for classification. Models such as the multi-layer perceptron (MLP) [33], deep neural network (DNN), support vector machine (SVM) [34], K-nearest neighbor (KNN) [35], Decision Tree (DT) [36], Random Forest (RF) [37], AdaBoost [38] and GradientBoosting [39] are potential classifiers. Since it is unknown in advance which model has the best classification performance, comparisons are needed before formally building Stage 2. Next, the classified 3D ROIs were input into the shared filter layer to remove the results with lower classification probabilities. Due to the multi-head 3D RPEs applied in Stage 1 as mentioned above, one same defect may be proposed as 3D ROIs by several different RPEs. Thus, the NMS [40] layer is introduced to remove redundant 3D ROIs. It calculates the intersection over union (IoU) values between 3D ROIs, and regards two 3D ROIs with IoU greater than a specified threshold as overlapping, then removes the 3D ROI with a smaller classification probability and keeps only the 3D ROI with a larger classification probability, and loops in this way until there are no overlapping 3D ROIs. Finally, the segmented defects are produced.

#### 3.2.3. Outputs of Mask-Point

At the end of Mask-Point, the geometric features CHA, CHV, OBBL, OBBW, and OBBD can be output. The body center coordinates OBBCX, OBBCY, and OBBCZ of OBB can also be extracted in the GGFE layer, which can provide data support for the quantitative evaluation of surface defects and information for the subsequent quantitative repair process of surface defects. OBBL, OBBW, and OBBD provide dimensional information on surface defects that can be used to assess the quality of the RTM process. OBBCX, OBBCY, and OBBCZ report the spatial coordinates of the defects, and the coordinates can be used by automated repairing tools to locate the spatial position of the defects. CHV and CHA provide the reference for the epoxy resin injection volume in the subsequent automated repair process. In summary, the Mask-Point in this paper can provide technical support for the intelligent manufacturing process of FRRMC products.

### 3.3. Distributed Surface Defects Detection System with Mask-Point

**Figure 10:**
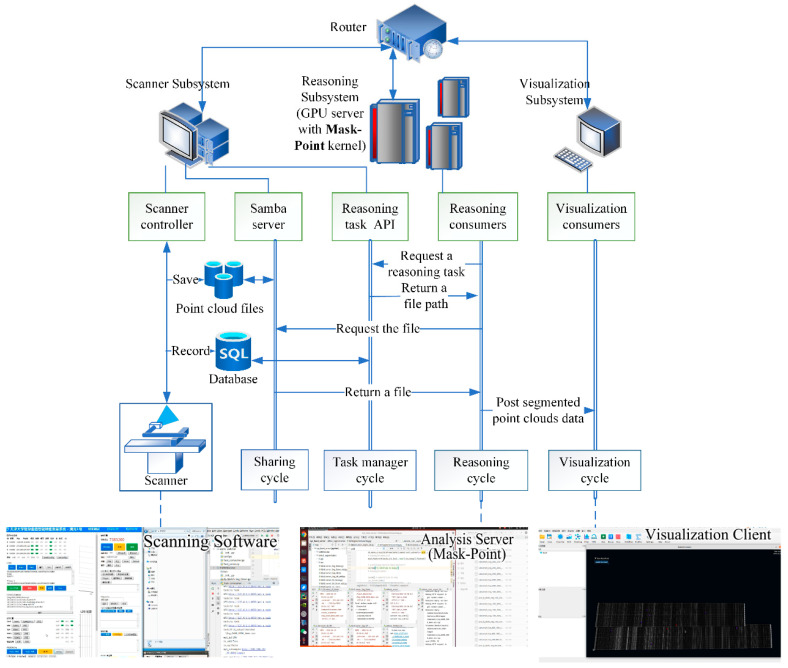
The architecture of a distributed surface defects detection system with Mask-Point for the manufacturing process of real FRRMC products.

A distributed and surface defects detection system is developed in the laboratory based on Mask-Point for the manufacturing process of real FRRMC products. The system’s architecture is shown in Figure 10, and it consists of three subsystems: the scanner subsystem, the inference subsystem, and the visualization subsystem. Each subsystem can run its own task and communicate with each other through a local area network. The scanner subsystem scans FRRMCs parts while storing the point cloud files and recording them into the database; it provides the reasoning API and Samba Server [41] to allow the reasoning subsystem to request the defect detection tasks and corresponding point cloud files. The reasoning subsystem keeps executing the following loops: request a reasoning task, obtain the task path, fetch the point cloud file according to the task path, execute the defects detection with Mask-Point kernel, and send the segmented results to the visualization subsystem. The visualization subsystem keeps executing the cycle of rendering. The visualization subsystem is established on the advanced Vulkan graphics API [42]; thus, the rendering frame rates keep above 20FPS even with 100 million points. The different subsystems are decoupled from each other, and the inference subsystem can also be multiple opened in parallel across multiple processes, thus improving the detection efficiency of the system.

## 4. Results and Discussion

### 4.1. Training and Test Performance of Mask-Point

To verify the effectiveness of Mask-Point, the training set and test set made in Section 2 are used to train and test Mask-Point. The training procedures of the Mask-Point also require two stages corresponding to Mask-Point’s architecture: the shared classifier in Stage 2 is firstly trained, then, freezing the classifier, other parts of Mask-Point are trained.

For the shared classifier in Stage 2, seven machine learning models are compared because which model has the best performance is unknown in advance, including MLP [33], SVM [34], KNN [35], DT [36], RF [37], AdaBoost [38], and GradientBoosting [39]. All the above models are implemented by Scikit-learn [43], their pseudo-codes and parameters are listed in Table 3, and default values are adopted for parameters not specifically listed. A total of 4396 samples of 3D ROIs are obtained from Stage 1 with pre-defined parameters in total, 3077 (70%) samples are applied to train classifiers, and the remaining 1319 (30%) are used to test classifiers.

**Figure 11:**
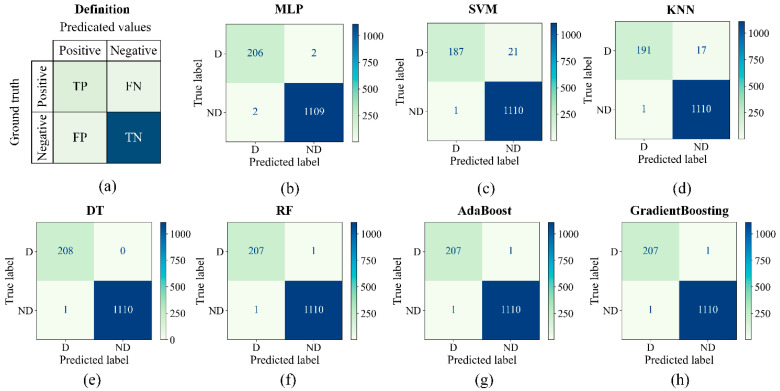
Seven confusion matrices of machine learning classifiers. (**a**) The definition of a confusion matrix; (**b**–**h**) confusion matrices of MLP, SVM, KNN, DT, RF, AdaBoost, and GradientBoosting, respectively.

Confusion matrices (see Figure 11) and several commonly used classification metrics (see Table 4) based on confusion matrices were introduced to evaluate the above classifiers’ performance. As shown in Figure 11a, the confusion matrix cross-tabulates predicted values and ground truths into four categories to summarize the performance of a binary classifier: (a) True Positive (TP): correctly predicting a label; (b) True Negative (TN): correctly predicting the other label; (c) False Positive (FP): falsely predicting a label; (d) False Negative (FN): missing and incoming label. Values with relatively larger TP and TN and smaller FN and FP indicate better performance. The confusion matrices of the seven classifiers are shown in Figure 11b–h, where label “D” represents defects and label “ND” represents non-defects.

The classification metrics, including the accuracy, precision, recall, f1 score, and *Matthews Correlation Coefficient* (MCC) [44], are introduced, and their definitions are listed in the table header of Table 4. The metrics reflect the performance of the classifier from different aspects. Larger values indicate better performance. From confusion matrices and metrics, it can be found that the DT model has the best performance with the largest metrics and the least FN and FP values. The sub-optimal models are RF, AdaBoost, and GradientBoosting models with the same confusion matrices. MLP’s performance is slightly worse than RF. These models above have the same level of performance indeed, as their MCC values differ by less than 0.01, and the difference between FN and FP values is less than two compared to thousands of samples; the small differences in performance may be caused by the splitting way of data or noises in engineering. SVM and KNN do not perform well, indicated by the large FN values. The MLP model is chosen as the shared classifier model in engineering because it has better scalability and can be accelerated in the GPU, and further fine-tuning of performance is also feasible, allowing Mask-Point to achieve high-speed inference as all layers of Mask-Point can run in the GPU if MLP is adopted. Of course, DT seems to be the best choice if only the CPU is provided, but its inference speed will be limited when dealing with massive data in practice.

With the trained classifier, other hyperparameters in the Mask-Point can be trained. Since the 3D RPEs in Stage 1 may affect the segmentation performance of the network, the performance of different numbers and parameters of 3D EPRs are compared. As shown in Table 5, one, two, and four different 3D RPEs are compared. Some of the key hyperparameters of Mask-Point are set as follows: the number of nearest neighbors of the grouping layer in the RPE is in array of [25, 30, 50, 75], the local geometric feature of the LGFE layer is NCR, and the parameter pairs (eps, minpts) of DBSCAN in the clustering layer are in [(0.15, 45), (0.125, 30), (0.175, 65), (0.2, 80)]. The suppression threshold of the NMS layer is 0.3. The commonly used evaluation metrics for the semantic segmentation task are the mAcc and the mIoU. mAcc is equal to the mean accuracy value of all categories, as shown in Equation (2). mIoU [45] of the segmentation calculates the ratio of the intersection over the union of the ground truths and the predicted result, as shown in Equation (3).

(2) $$\mathrm{mAcc}=\frac{{\mathrm{Acc}}_{\mathrm{defect}}+{\mathrm{Acc}}_{\mathrm{background}}}{2}$$

(3) $$\mathrm{mIoU}=\frac{1}{k+1}\overset{k}{\underset{i=0}{\sum }}\frac{p_{ii}}{\sum_{j=0}^{k}p_{ij}+\sum_{j=0}^{k}p_{ji}-p_{ii}}$$

where $\begin{array}{c} p_{ij} \end{array}$ represents the number of points whose true category is *i* but is predicted to be *j*. Similarly, $\begin{array}{c} p_{ji} \end{array}$ represents the number of points whose true category is *j* but is predicted to be *i*, and *k* + 1 is the total number of categories including empty categories. $\begin{array}{c} p_{ii} \end{array}$ represents the real quantity. As shown in Table 5, the mAcc and mIoU increase as the number of different 3D RPEs increases, indicating that the overall segmentation performance is improved with the number of different 3D RPEs increases. However, the inference speed is becoming slower because more 3D RPEs and subsequent networks in Stage 2 consume more computational resources. The Mask-Point with four 3D RPEs has the relatively best segmentation performance, and the corresponding accuracy, mIoU, and inference speed achieve 0.9997, 0.9402, and 320,000 points/s on a single NVIDIA RTX3090, respectively.

**Figure 12:**
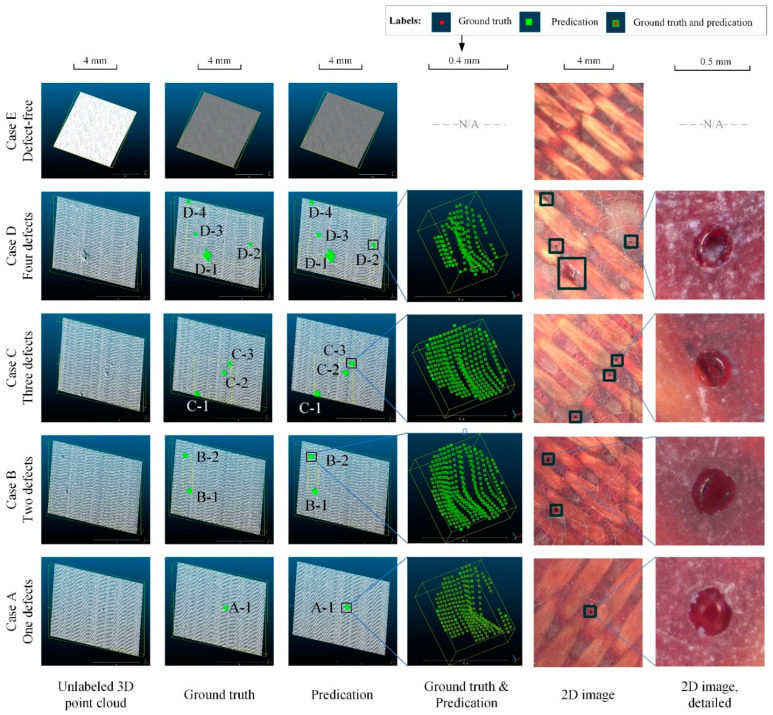
Three-dimensional surface defects detection cases on FRRMC test set with proposed Mask-Point. Where the labels like “A-1” indicates the number of the defect, the first letter indicates which case it belongs to, and the second number indicates its number. “N/A” indicates that the figure is not available because the corresponding case is defect-free.

Five detailed defects detection cases are shown in Figure 12. Cases A–D contain 1–4 surface defects, respectively. The original unlabeled 3D point clouds are listed in the first column, and the labeled ground truths are listed in the second column. The third column is the predicted values, and the fourth is ground truths and predictions in the same magnified view. The last two columns are corresponding 2D images. The macroscopic positions of the defects in predictions and ground truths correspond almost one-to-one, regardless of whether the sample has one or multiple defect instances, which intuitively demonstrates the good mAcc. In particular, the points in the red-centered green box in column 4 occupy almost all the points, and the points in the red-centered green box represent TP value points in the confusion matrices, indicating that the predicted points almost overlap with the ground truths. There are also a few scattered green points in the fourth column, which indicate that Mask-Point segments out more points than ground truths, indicating that the algorithm in this paper is broader than ground truths to prevent underestimation. Case 5 is a defect-free instance, as Mask-Point does not misclassify any point as a defect category. The above performance shows that surface defects of FRRMCs can be accurately and efficiently segmented from the 3D point cloud with Mask-Point.

**Figure 13:**
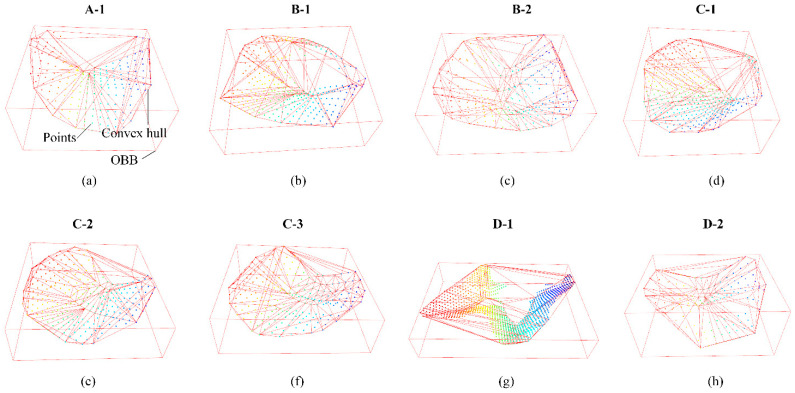
Convex hulls and OBBs of some surface defects regarding Figure 12 computed from predictions. (**a**) The convex hull and OBB of A-1; (**b**) the convex hull and OBB of B-1; (**c**) the convex hull and OBB of B-2; (**d**) the convex hull and OBB of C-1; (**e**) the convex hull and OBB of C-2; (**f**) the convex hull and OBB of C-3; (**g**) the convex hull and OBB of D-1; (**h**) the convex hull and OBB of D-2.

The outputs of Mask-Point are shown in Table 6, including the global geometric features and center coordinates of each surface defect corresponding to Figure 12. The same features computed from ground truths are listed in Table 7. Some of the corresponding convex hulls and OBBs are shown in Figure 13. It can be seen that the difference between the predicted and ground truth values of the OBB series values and CHA is small, and only some of the CHV values are slightly different. For example, the predicted CHV of defect D-1 is 5.1993 mm^3^, while the ground truth CHV is 3.6478 mm^3^. Different boundary points of ground truths and predications lead to different convex hulls, thus affecting the estimation of the volume value. More accurate CHV estimation may rely on finely tuned scanning parameters and will be explored in future works.

### 4.2. Comparison of Mask-Point and Typical 3D Semantic Segmentation Networks

**Figure 14:**
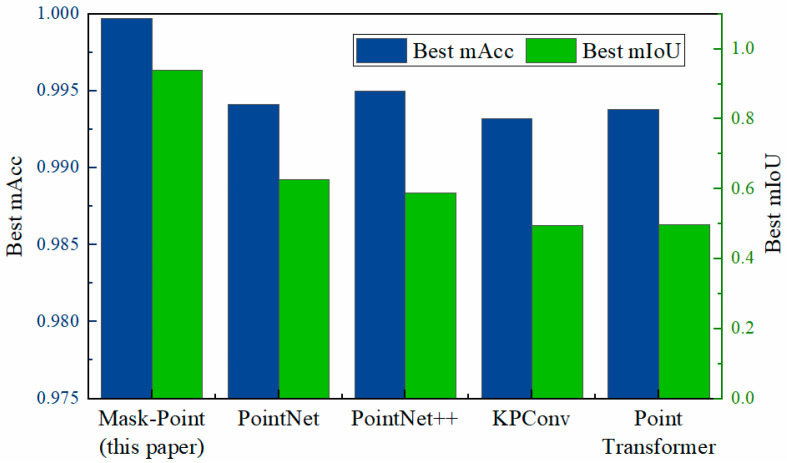
Comparison of Mask-Point with several other typical semantic segmentation networks on the test set.

To further verify the performance of Mask-Point, several other typical 3D semantic segmentation networks are preliminarily compared, including PointNet [14], PointNet++ [15], KPConv [46], and PointTransformer [16]. The PointNet and PointNet++ are implemented from Xu’s repository [47], and the KPConv and PointTransformer implemented from Open3D-ML’s repository [48]. All the parameters concerning the number of samples were set to 160,000 if needed, i.e., all points were forced to participate in the training and test. The other parameters were improved mainly based on the default parameters but with little changes. The networks were trained and tested on the same GPU server with an NVIDIA RTX 3090 using the FRRMCs dataset made above. The best results of each model trained with different parameters are shown in Table 8 and Figure 14. Mask-Point achieves the relatively best performance compared with other networks. The accuracy and mIoU reached 0.9997 and 0.9402, respectively. The mIoU of Mask-Point is increased by about 30% compared with the sub-optimal network, i.e., PointNet.

The relatively poor performance on mIoU of other networks may be due to the irregular shape of the surface defects, making it more difficult for the networks to extract general features. In addition, other networks belong to the one-stage networks and do not have the 3D RPEs-like structures. So, it may be difficult for these networks to locate defect regions from a large number of point clouds, especially when the sizes of defects are significantly smaller than the backgrounds and the points’ number of defects is significantly less than the backgrounds with limited samples. Conversely, the 3D RPEs in Stage 1 of Mask-Point could generate different 3D ROIs, allowing Stage 2 to focus on the potential defect regions, thus finally performing a relatively high defects segmentation performance. Mask-Point provides a new possible method for accurate surface defects detection of FRRMCs.

### 4.3. Verification of Mask-Point

**Figure 15:**
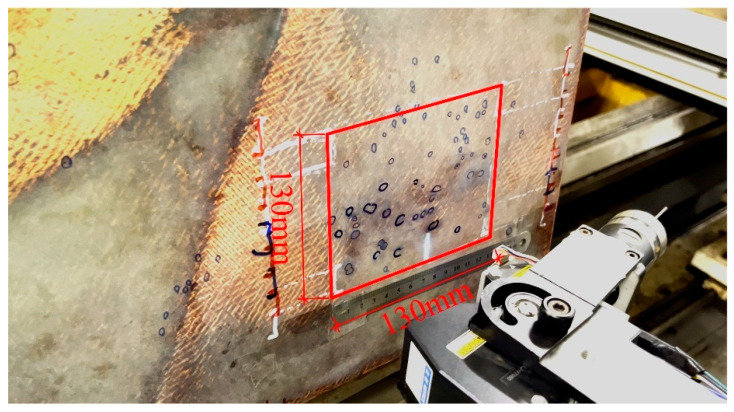
In a real FRRMC product, the area inside the red frame is used for assessing the performance of the proposed distributed surface defects detection system with Mask-Point.

**Figure 16:**
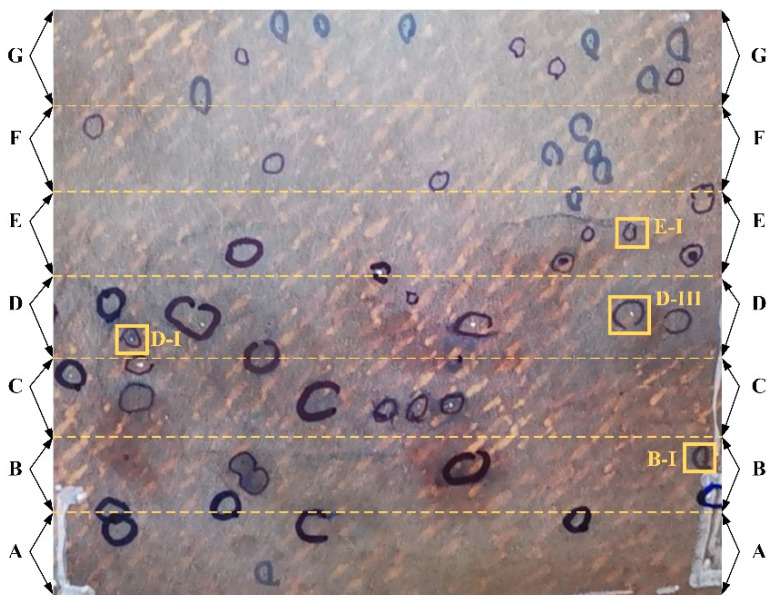
Ground truths which are labeled by the most skilled human workers and human experts. The labels B-I, D-I, D-III, E-I are individually marked defects for comparison.

In addition to the above FRRMC dataset-based experiments, the proposed Mask-Point is also applied to detect surface defects of real FRRMC products using the proposed distributed surface defects detection system mentioned in Section 3 and compared with skilled human workers. A real FRMMC product is shown in Figure 15. The area to be examined is framed in red. The Mask-Point-based system accomplishes scanning and defects detection within five minutes. Two skilled workers from the front line are engaged to detect defects individually as many as possible within five minutes. Ground truths are determined by the most skilled human workers and experts with unlimited time, as shown in Figure 16. The examined areas were divided into seven from Area A to G.

**Figure 17:**
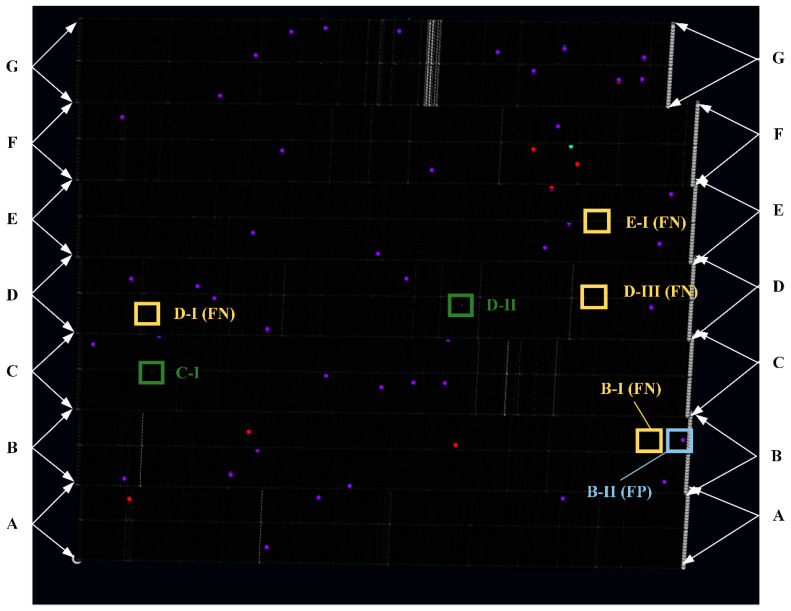
Screenshot of detected defects in the self-developed visualization software with 26.63 million points. Each colored dot represents an instance of the detected defect. B-I, D-I, D-III, and E-I belong to FN. B-II belongs to FP; C-I and D-II are correctly detected defects but not obvious, so they are marked to avoid confusion for readers.

The results scanned and detected by the Mask-Point system are shown in Figure 17. It contains about 26.63 million points in total, and each colored point represents a defect instance. The comparison of human workers and Mask-Point is listed in Table 9. It can be seen that skilled workers can recognize most of the surface defects, and their accuracies and recalls are close to 0.9, F1 scores are close to 0.94, and FPs are 0, indicating that humans do not recognize non-defects as defects. However, the human workers take more time to adjust the light and the position of the magnifier during the detection and therefore cannot achieve high performance within the limited 5 min. They believe that they can achieve better performance if more time is allowed. Mask-Point achieves the relatively best performance compared with human workers in the same time limit with precision, accuracy, f1 score, and recall values of 0.9630, 0.9643, 0.9811, and 0.6939, respectively.

However, there are also four FNs (see yellow boxes in Figure 17) and one FP (see the blue box in Figure 17) produced by Mask-Point. Four FNs (B-I, D-I, D-III, E-I) are found at the beginning and end of Area B, D, and E. The FNs and FP may be due to insufficient geometric features of the defects or insufficient acquisition of the original data caused by the low scanning frequency. The FP (B-II) is found to be caused by a piece of dust that was raised on the surface. Because the current Mask-Point uses only the basic features of the OBB, their orientation relative to the entire surface is not considered, i.e., whether 3D ROIs are raised or concave is not identified by Mask-Point, and therefore, the misjudgment is produced. The misjudgment can be avoided by surface cleaning before engineering inspection, and the algorithm’s improvement will be carried out in future works. Besides, it is also worth noting that C-I and D-II in Figure 17 are correctly detected defects but not obvious, so they are marked by green boxes to avoid confusion for readers. These experiments proved that the proposed distributed surface defects detection system with Mask-Point outperformed the skilled workers in a limited time.

### 4.4. Summary of Experiments

Three experiments, including training and validation experiments, comparison experiments, and verification experiments, are studied in this section. The first two experiments were performed on the FRRMCs dataset produced in this paper. The verification experiments were performed on the real FRRMC products.

In the training and test experiments, seven different machine learning models were compared to find a better classifier in Stage 2 of Mask-Point. The MLP model was chosen because of its good performance, and it can be accelerated by GPU and facilitate parallel computation. Different types and numbers of 3D RPEs were also compared because they affect the overall performance of Mask-Point. The Mask-Point with four 3D RPEs achieves the relatively best segmentation performance, and the corresponding accuracy, mIoU, and inference speed reach 0.9997, 0.9402, and 320,000 points/s on a single NVIDIA RTX3090, respectively. A comparison of ground truths and predictions is shown in Figure 12, which visually demonstrates the high mIoU achieved by the proposed method. In addition, the predicted OBB series features, CHA, and CHV are also output to support the subsequent repair process.

To further verify the performance of Mask-Point, several other typical 3D semantic segmentation networks are preliminarily compared. The mIoU of Mask-Point is about 30% ahead of the sub-optimal 3D semantic segmentation network PointNet. A Mask-Point-based distributed surface defect detection system is developed and applied in the scanning and defect detection of real FRRMC products. Mask-Point achieves the relatively best precision, accuracy, f1 score, and recall values of 0.9630, 0.9643, 0.9811, and 0.6939 in competitions with skilled human workers within limited five minutes. It is generally 1% to 3% ahead of the skilled human workers. The above three experiments demonstrate that Mask-Point can accurately and efficiently detect surface defects in FRRMCs.

In addition, there are still many aspects of Mask-Point can be improved. It can be observed that the integrity of 3D point clouds is less than 2D images, as shown in the fourth and sixth columns of Figure 12. The predicted CHV and the CHV of the ground truths are slightly different in Table 6 and Table 7. The above problems may be related to the 3D scanner and the scanning process. The scanning process parameters and the filtering techniques thus deserve further studies. A piece of raised dust is found to be the cause of the FP misclassification, as shown in Figure 17 B-II. The classifier in Mask-Point should also be further upgraded to determine whether the 3D ROIs are raised or recessed to improve the defect detection performance further.

## 5. Conclusions

In order to accurately and efficiently detect the 3D surface defects of FRRMCs, a novel two-stage 3D semantic segmentation network especially designed for the surface defects detection task, i.e., Mask-Point, is proposed. Stage 1 of Mask-Point generates many 3D ROIs, allowing subsequent networks to focus on potential regions of defects. Stage 2 classifies and aggregates the 3D ROIs; the two stages together lead to relatively better segmentation results. To evaluate the performance of the Mask-Point, a new 3D surface defects dataset of FRRMCs containing about 120 million points is produced. The following conclusions are obtained:

(a) Multi-head 3D RPEs in Stage 1 make the Mask-Point can deal with multi-density data in engineering, overcome the sensitivity to hyperparameters, and facilitate parallel computing. The shared classifier in Stage 2 of Mask-Point with features CHA, CHV, OBBL, OBBW, and OBBD can effectively classify 3D ROIs.
(b) Training and test experiments show that the accuracy and mIoU increase as the number of different 3D RPEs increases, but the inference speed becomes slower when the number increases. The Mask-Point with four 3D RPEs has the relatively best segmentation performance; the corresponding accuracy, mIoU, and inference speed achieve 0.9997, 0.9402, and 320,000 points/s on a single NVIDIA RTX3090, respectively.
(c) Preliminary comparison experiments also indicate that Mask-Point offers relatively best segmentation performance compared with several other typical networks. The mIoU of Mask-Point is about 30% ahead of the sub-optimal 3D semantic segmentation network PointNet.
(d) A Mask-Point-based distributed surface defect detection system is developed. The system is applied to scan real FRRMC products and detect their surface defects. It achieves the relatively best precision, accuracy, f1 score, and recall values of 0.9630, 0.9643, 0.9811, and 0.6939 in competitions with skilled human workers within limited five minutes. Mask-Point is generally 1% to 3% ahead of the skilled human workers.

The above experiments demonstrate that the proposed Mask-Point cloud relatively accurately and efficiently detect surface defects of FRRMCs. It offers new potential technology support for defect-free manufacturing of FRRMCs. Mask-Point is also applicable to the other surface defects detection tasks with other similar materials rather than only FRRMCs as long as their surfaces have similar characteristics to resin materials. In the future, a larger dataset will be produced to improve the performance of Mask-Point further, and more in-depth research will be conducted.

## Figures and Tables

**Table 1:** Technical specification of the XYZ robot.

Item	Value
Range	X: 0–1000 mm, Y: 0–550 mm; Z: 0–300 mm
Repeatability	X, Y, and Z: ±3 µm/m (Linear encoders)

**Table 2:** Technical specification of the laser displacement sensor.

Model			LJ-V7060
Reference distance			60 mm
Measurement range	*z*-axis (height)		±8 mm (F.S. = 16 mm)
	*x*-axis (width)	NEAR side	13.5 mm
		Reference distance	15 mm
		Far side	
Repeatability	*z*-axis (height)		0.4 µm
	*x*-axis (width)		5 µm
Linearity	*z*-axis (height)		±0.1% of F.S.
Profile data interval	*x*-axis (width)		20 µm
Sampling cycle (trigger interval)			Top speed: 16 µs (high-speed mode)

**Table 3:** Seven machine learning models for the shared classifier and their pseudo-codes (Scikit-learn [43] style).

Model	Parameters
MLP	MLPClassifier(hidden_layer_sizes = (32.64), max_iter = 500)
SVM	svm.SVC (probability = True)
KNN	neighbors.KNeighborsClassifier()
DT	tree.DecisionTreeClassifier()
RF	RandomForestClassifier(n_estimators = 50)
AdaBoost	AdaBoostClassifier(n_estimators = 50)
GradientBoosting	GradientBoostingClassifier(n_estimators = 50, learning_rate = 1.0, max_depth = 1)

**Table 4:** Performance of seven classifiers.

Metric	Accuracy	Precision	Recall/Sensitivity	F1 Score	Matthews Correlation Coefficient (MCC)
	ACC = (TP + TN)/(P + N)	PPV = TP/(TP + FP)	TPR = TP/(TP + FN)	F1 = 2TP (2TP + FP + FN)	MCC = TP × TN − FP × FN/sqrt((TP + FP) × (TP + FN) × (TN + FP) × (TN + FN))
MLP	0.9970	0.9904	0.9904	0.9904	0.9886
SVM	0.9833	0.9947	0.8990	0.9444	0.9363
KNN	0.9864	0.9948	0.9183	0.9550	0.9482
DT	0.9992	0.9952	1.000	0.9976	0.9972
RF/ AdaBoost/GradientBoost	0.9985	0.9952	0.9952	0.9952	0.9943

**Table 5:** Comparison of different numbers of different 3D RPEs on the FRRMC test set.

Number of Different 3D RPEs	mAcc	mIoU	Inference Speed (Points/s)
1	0.9462	0.6328	410,000
2	0.9698	0.8269	350,000
4	0.9997	0.9402	320,000

**Table 6:** Global geometric features and center coordinates of each surface defect in Figure 12 computed from predictions.

Name	Number	OBBCX /mm	OBBCY /mm	OBBCZ /mm	OBBD /mm	OBBW /mm	OBBL /mm	CHV /mm^3^	CHA /mm^2^
A-1	233	78.2883	19.3031	58.5207	0.1893	0.3693	0.3837	0.2767	0.2488
B-1	314	79.0784	13.1296	59.8993	0.3081	0.4613	0.4873	0.1825	0.4077
B-2	304	78.6286	12.9802	63.6387	0.1778	0.3877	0.4217	0.4421	0.2763
C-1	396	30.6412	10.8558	57.2657	0.2017	0.4417	0.4574	0.7670	0.3830
C-2	283	33.1384	10.8632	60.2893	0.1934	0.3885	0.4177	0.5223	0.2905
C-3	255	33.6745	10.8470	61.4207	0.1149	0.3422	0.3908	0.0837	0.2144
D-1	1147	48.2271	11.4217	67.1759	0.3800	0.8790	1.3698	5.1993	1.7152
D-2	164	52.4099	11.4485	69.5911	0.1621	0.2424	0.3141	0.2983	0.1773
D-3	197	47.0998	11.2117	69.2650	0.1640	0.2799	0.3125	0.3461	0.2125
D-4	165	46.3314	11.0236	72.7422	0.1351	0.2483	0.3471	0.0515	0.1475

**Table 7:** Global geometric features and center coordinates of each surface defect in Figure 12 computed from ground truths.

Name	Number	OBBCX /mm	OBBCY /mm	OBBCZ /mm	OBBD /mm	OBBW /mm	OBBL /mm	CHV /mm^3^	CHA /mm^2^
A-1	228	78.2882	19.3035	58.5199	0.1888	0.3333	0.3842	0.1558	0.2483
B-1	305	79.0781	13.1304	59.8964	0.2992	0.4532	0.4631	0.0616	0.3969
B-2	301	78.6285	12.9807	63.6382	0.1760	0.3381	0.4053	0.4940	0.2746
C-1	391	30.6416	10.8565	57.2661	0.2023	0.4420	0.4628	0.5529	0.3784
C-2	271	33.1422	10.8642	60.2898	0.1939	0.3795	0.4147	0.1628	0.2837
C-3	243	33.6756	10.8483	61.4182	0.1088	0.3420	0.3807	0.1361	0.2059
D-1	1037	48.2236	11.4240	67.1782	0.3947	0.8316	1.2739	3.6478	1.6350
D-2	154	52.4138	11.4492	69.5929	0.1577	0.2347	0.3181	0.3031	0.1754
D-3	185	47.1024	11.2122	69.2642	0.1705	0.2865	0.2947	0.2356	0.2049
D-4	158	46.3312	11.0248	72.7408	0.1345	0.2440	0.3235	0.0519	0.1431

**Table 8:** Comparison of Mask-Point with several other typical semantic segmentation networks on the test set.

Model	Mask-Point (This Paper)	PointNet	PointNet++	KPConv	PointTransformer
Best mAcc	0.9997	0.9941	0.9950	0.9932	0.9938
Best mIoU	0.9402	0.6272	0.5881	0.4953	0.4983

**Table 9:** Comparison of human workers and Mask-Point with the same limited five minutes.

Region/Metric	Ground Truth	Human Worker 1			Human Worker 2			Mask-Point		
		TP	FN	FP	TP	FN	FP	TP	FN	FP
A	4	4	0	0	4	0	0	4	0	0
B	7	6	1	0	7	0	0	6	1	1
C	8	6	2	0	7	1	0	8	0	0
D	9	7	2	0	7	2	0	7	2	0
E	8	8	0	0	8	0	0	7	1	0
F	7	6	1	0	7	0	0	7	0	0
G	11	10	1	0	8	3	0	11	0	0
In total	54	47	7	0	48	6	0	50	4	1
Precision	-	1.000			1.000			0.9804		
Accuracy		0.8704			0.8889			0.9091		
F1 Score		0.9307			0.9412			0.9524		
Recall		0.8704			0.8889			0.9259		

## Data Availability

Not applicable.
